# Temporal trend and spatial analysis of the HIV epidemic in young men who have sex with men in the second largest Brazilian Amazonian province

**DOI:** 10.1186/s12879-022-07177-w

**Published:** 2022-02-24

**Authors:** Iaron Leal Seabra, Andrey Oeiras Pedroso, Taymara Barbosa Rodrigues, Glenda Roberta Oliveira Naiff Ferreira, Ana Lucia da Silva Ferreira, Ricardo Alexandre Arcêncio, Dulce Gomes, Richardson Augusto Rosendo da Silva, Eliã Pinheiro Botelho

**Affiliations:** 1 Universidade Federal do Pará, Programa de Pós-Graduação em Enfermagem, Rua Augusto Correia, 01 – Setor saúde, GuamáBelém, 66075-110 Pará Brasil; 2 Departamento de Vigilância Epidemiológica, Secretaria de Saúde Pública do Pará, Av. Doutor Freitas, 235 Sacramenta, 66123-050 Belém, Pará Brasil; 3 Maternal-Infant and Public Health Nursing Departament, University of São Paulo, College of Nursing at Ribeirão Preto, Avenida Dos Bandeirantes, 3900 - Campus Universitário, Monte Alegre, 14040-902 Ribeirão Preto, São Paulo, Brasil; 4 Departamento de Matemática, Colégio Luís António Verney, Universidade de Évora, Rua Romão Ramalho,, 597000-671 Évora, Portugal; 5 Departamento de Enfermagem, Centro de Ciências da Saúde,, Universidade Federal do Rio Grande do Norte, Rua General Cordeiro de Faria, S/N. Petrópolis, 59012-570 Natal, Rio Grande do Norte, Brasil

**Keywords:** Social determinants of health, HIV, Youth, Spatiotemporal analysis

## Abstract

**Background:**

After 40 years of its starting, the HIV epidemic in Brazilian Amazon region remains on an increasing trend. The young men who have sex with men (MSM) have been the most impacted by the HIV in the last decade. However, much more than attributing the risk behavior to HIV uniquely to the individual, behaviors are shaped by social determinants of health (SDH). Despite the problem, there is a scarcity of studies evaluating the impact of SDH on HIV among young MSM and none of them were done in the Northern of Brazil. Therefore, the main goal of this study was to analyse the HIV epidemic among Brazilian Amazonian young MSM using temporal trends and spatial analysis.

**Methods:**

We conducted an ecological study using reported cases of HIV/AIDS in young MSM living in Pará, the second larger Brazilian Amazonian province, between 2007 and 2018. Data were obtained from the Information System for Notifiable Diseases. For the temporal analysis, we employed a Seasonal and Trend decomposition using Loess Forecasting model (STLF), which is a hybrid time-series forecast model, that combines the Autoregressive-Integrated Moving Average (ARIMA) forecasting model with the Seasonal-Trend by Loess (STL) decomposition method. For the spatial analysis, Moran’s spatial autocorrelation, spatial scan, and spatial regression techniques were used.

**Results:**

A total of 2192 notifications were included in the study. Greater variabilities in HIV/AIDS population-level diagnosis rates were found in the festive months. The HIV/AIDS population-level diagnosis rates exhibited an upward trend from 2013 and this trend is forecasted to continue until 2022. Belém, the capital of Pará, presented the highest spatial risk for HIV/AIDS and was the only city to present spatiotemporal risk from 2014 to 2018. The geographic variation of the HIV epidemic was associated with the number of men with formal jobs, the average salary of men, and the percentage of people over 18 years old with elementary education.

**Conclusion:**

The upward trend of HIV/AIDS population-level diagnosis rate forecasted until 2022 and the variability of the epidemic promoted by the SDH brings an alert and subsidies to health authorities to implement more efficient and focalized public policies against HIV among young MSM in Pará.

## Background

In the last decade, young people have been highly impacted by the HIV epidemic. Approximately 30% of newly detected HIV cases worldwide are among young people [1]. In Brazil, the greatest increase in HIV detection rates occurs among young men, with most of them included in the men who have sex with men (MSM) group exposure category [2]. This group of young MSM is more likely to be infected with HIV than young heterosexual individuals. This phenomenon may be related to the earlier initiation of sex among young MSM, multiple sexual partners, social stigmatization as well as other social and behavioral factors that make them more vulnerable to HIV than young heterosexual men [3, 4]. According to the Brazilian Youth Statute, a person aged between 15 and 29 years is considered a young person [5]. Only in 2019, Brazil recorded 26,141 cases of HIV/AIDS among men with a predominance of cases in MSM exposure group compared to the heterosexual one (39.85% vs. 39.5%, respectively). However, in the Northern of Brazil the heterosexual group had a small predominance compared with the MSM group, from 2322 cases of HIV/AIDS notified among men, about 45% were in MSM and about 48% in heterosexual men [2].

However, risk behaviors to HIV are shaped by social determinants of health (SDH) and cannot be attributed uniquely to the individual. SDH are the local conditions in which people live. They are comprised of cultural, social, and economic aspects which can lead to the occurrence of disease and risk factors [6]. Therefore, SDH confers a distinct identity on the HIV epidemic in each specific territory. In Brazil, factors associated with poverty, such as low income and low schooling, as well as the stigma against HIV and gays have been pointed as directedly associated with the HIV-positive status among young MSM [7, 8].

Among all Brazilian regions, the Brazilian Amazon is the poorest one with a large part of its population living in precarious conditions of housing and basic sanitation, low education level, low coverage of health services and low monthly wage [9]. These factors can explain the observed HIV epidemic scenery in this region, where in the last decade the HIV/AIDS detection rate increased by 24.4%. Among the states that comprise the northern region, Pará had the second-highest increase in the HIV/AIDS detection rate (46.5%). Among the Brazilian federative units and capitals, Pará and its’ capital Belém, stood out as the fourth and second in HIV/AIDS detection rates rank, respectively [2].

Spatial and temporal analysis techniques are useful tools for providing an enhanced understanding of the HIV epidemic. Spatial analysis allows the visualization of regions with the highest epidemiological pressure and associates the phenomenon with SDH. In comparison, temporal analysis provides insight into the epidemic’s behavior over time. It also provides an evaluation of the impact of implemented public policies designed to control the epidemic [10, 11].

Despite the identified problem, a literature review with the descriptors “HIV”, “young,” “men who have sex with men” and “spatial analysis” resulted in no studies. In addition, considering the impact of HIV in the young MSM and the epidemiological scenery in the Brazilian Amazon region, it is necessary to evaluate how the implemented public policies to fight HIV are impacting the young MSM and to comprehend the epidemic geographical variability promoted by the SDH to implement more focalized and efficient public policies against HIV in this province.

Therefore, we aimed to analyze the HIV epidemic among Brazilian Amazonian young MSM employing temporal trend and spatial analysis in Pará, Brazil. For the temporal analysis we employed the Seasonal and Trend decomposition using Loess Forecasting model (STLF) method For spatial analysis, we used Moran’s spatial autocorrelation, spatial scan, and spatial regression.

## Methods

### This was an ecological study

#### Study design and population

**Fig. 1:**
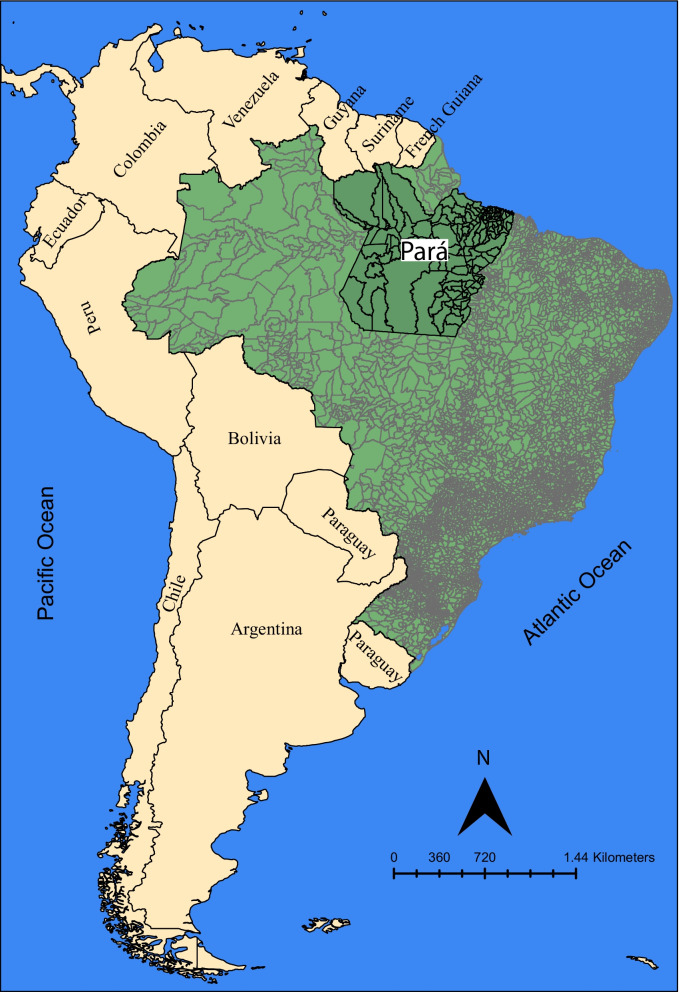
Spatial location of Brazil (light green) in South America continent and of Pará

Pará is in the northern region of Brazil and is the second-largest state in terms of territorial area (1,245,870,707 km^2^). It is divided into 144 municipalities with a population of 8,690,745 inhabitants [12] (Fig. 1). Despite its abundance of natural resources, the state has the third-lowest Brazilian Human Development Index (HDI = 0.698), high income inequality distribution (Gini = 0.53), and a low coverage rate of primary healthcare services (59.13%). The majority of the primary healthcare places (68.2%) are in urban areas. Thus, people living in rural population zones have difficulty accessing health services [13].

The study population consisted of HIV/AIDS cases among young MSM living in Pará. These cases were recorded in the Information System for Notifiable Diseases between 2007 and 2018. All data were provided by the State Department of Public Health in Pará. Only notifications containing the city of residence and age of the individual were included in the study. The following variables were collected: age, year of diagnosis, race, educational level, and city of residence. Data were double-checked and redundant and inconsistent data were removed.

### Data analysis

Descriptive analysis was performed using Microsoft Office Excel 365® 2019 (Microsoft Corporation, Santa Rosa, CA, USA). The results were expressed as absolute values (n) and relative frequencies (%).

For the temporal analysis, we used the monthly HIV/AIDS population-level diagnosis rates following previous study [14]. To the calculus of the population-level diagnosis rates, the number of cases reported in a specific month in young MSM in Pará was divided by the population projection of all young men aged 15–29 in the specific year living in Pará due to the number of MSM are not available in Brazil. It was then multiplied by 100,000 inhabitants.

The temporal analysis was done employing a Seasonal and Trend decomposition using Loess Forecasting model (STLF), a hybrid time-series forecast model, which combines the Autoregressive-Integrated Moving Average (ARIMA) [15] forecasting model with the Seasonal-Trend by Loess (STL) decomposition method [16]. The great advantage of this new approach is the high capacity to make accurate long-term forecasts, contrary to the isolated use of ARIMA models, whose long-term forecasts tend to the average of the series, therefore unrealistic.

The STL method is a filtering method that consists of decomposing a time series as a sum of three components of variation: seasonality, general trend and remainder. This decomposition is based on robust local regression, employing a locally weighted regression (Loess), of trend and seasonal components. On the other hand, the STLF method considers the decomposition of the series into three components and, from the three estimated components, uses this information to predict the future values of the series. To this end, it considers that the seasonality pattern remains the same in the coming years and takes together the trend and the error (usually called seasonally adjusted data). An ARIMA model is fitted to this combination and, based on this, the corresponding forecasts are made.

Before proceeding to the modelling of the series, and the consequent prediction of future values, possible structural changes in the series were investigated using the method developed by Zeileis et al. [17]. All analyses were performed on RStudio software (Version 1.4; RStudio, Boston, MA, USA) using the strucchange [17] and forecast [18] R packages.

In the spatial analysis, the spatial distribution and autocorrelation of the HIV/AIDS population-level diagnosis rates were determined using ArcGIS 10.1, software (ESRI, Redland, California, United States). The municipalities HIV/AIDS population-level diagnosis rates were calculated for quadrenniums (2007–2010, 2011–2014, and 2015–2018) and for the entire period of the study to avoid annual fluctuations. The calculation of the population-level diagnosis rates was based on the average population projections of young men in the municipalities for each specific period [14]. Spatial autocorrelation of the HIV epidemic among young MSM was analyzed using Moran’s global index (*I*) with 999 permutations. This was followed by the statistical method and local indicators of spatial association (LISA). A queen-type W contiguity matrix was employed in both analyses and neighboring municipalities were considered to share borders and nodes.

*I* index ranges from − 1 to 1, where 1 indicates a direct correlation, 0 indicates randomness, and − 1 indicates an inverse correlation. Four types of clusters can be identified in the LISA map: high–high and low–low indicates a direct correlation, whereas low–high and high–low indicates an inverse correlation.

Spatial scan analysis based on the discrete Poisson model was performed using the SatScan 9.7 software (Kulldorf, Cambridge, MA, USA) to assess the regions at risk for HIV/AIDS [19]. The following criteria were used for the spatial risk detection: non-overlapping clusters with a maximum size of 50% of the population at risk and 999 replications. The same criteria were used for the spatio-temporal risk detection, however considering the maximum size of the temporal cluster as equal to 50% of the study period. Each risk region had a relative risk (RR) and 95% CI. Regions with RR ≥ 1 and *p* ≤ 0.05, were considered at risk. RStudio 1.4 software was used to calculate the 95% CI.

The geographic variability of the HIV/AIDS epidemic in Pará promoted by SDH was assessed using geographically weighted regression (GWR) analysis [20]. The HIV/AIDS population-level diagnosis rate for the 12 years of the study was considered a dependent variable. The SDH were the independent variables.

The SDH projected for 2018, based on 2000 and 2010 census data, were obtained from websites of the Institute of Applied Economic Research and Brazilian Institute of Geography and Statistic. They were then categorized into the following dimensions: Education: (1) percentage of people aged 18 or over without complete primary education and with informal work, (2) elementary school dropout rate, (3) high school dropout rate, (4) percentage of people aged 18 or over with complete primary education, and (5) school attendance; Income: (1) people who live in households with per capita income less than the minimum wage and require more than an hour to arrive at work, (2) percentage of people between 15 and 24 years of age who do not study or work and have a per capita income equal to or less than the minimum wage, (3) proportion of people with per capita income less than or equal to half the minimum wage, (4) unemployment rate of the population of 18 years of age or over, (5) total number of families registered in the *Bolsa Família* Programme, (6) total number of families registered in Single Register of Social Programmes, (7) formal employment relationships—male, and (8) average salary of formal workers—male; Social prosperity: (1) municipal HDI (HDIm), HDIm-longevity, (3) HDIm-education, (4) HDIm-income, and (5) the social prosperity index; Primary Care Coverage: Environmental and Social Framework coverage.

For GWR, Pearson's correlation analysis was used to verify the correlation between the dependent and independent variables in RStudio. All correlations (*p* ≤ 0.05) were then analyzed through an ordinary least squares (OLS) regression model using the step-by-step method in MGWR software (ASU, MD, USA). The generated models were evaluated for multicollinearity and only those with variance inflation factor (VIF) values lower than 10 were accepted. The best explanatory model for this phenomenon was defined by the Akaike information criterion (AIC) value (*p* < 0.05). The OLS residuals were then analyzed for spatial autocorrelation to validate the model. After the spatial dependence of the residuals was discarded, GWR was applied considering the adaptative-bandwidth kernel since it had the smaler AIC when compared with the fixed-bandwidth (Adaptative: 654.504; Fixed: 654.774). The GWR residuals were also analyzed for spatial autocorrelation. The adjusted R^2^, AIC, and corrected Akaike information criterion (AICc) values obtained from the GWR were used to compare the OLS and GWR models.

## Results

A total of 2220 HIV/AIDS cases were reported among young MSM in Pará between 2007 and 2018. However, only 2192 notifications were included in the study due to missing data. The average age of the young people notified was 23.6 ± 2.12 years, with most notifications in the age group of 20–24 years (n = 995, 45.39%). Most young people declared themselves as black (n = 1714, 78.19%), with a high school education (n = 875, 39.92%), and as living in urban areas (n = 2011, 91.74%).

**Fig. 2:**
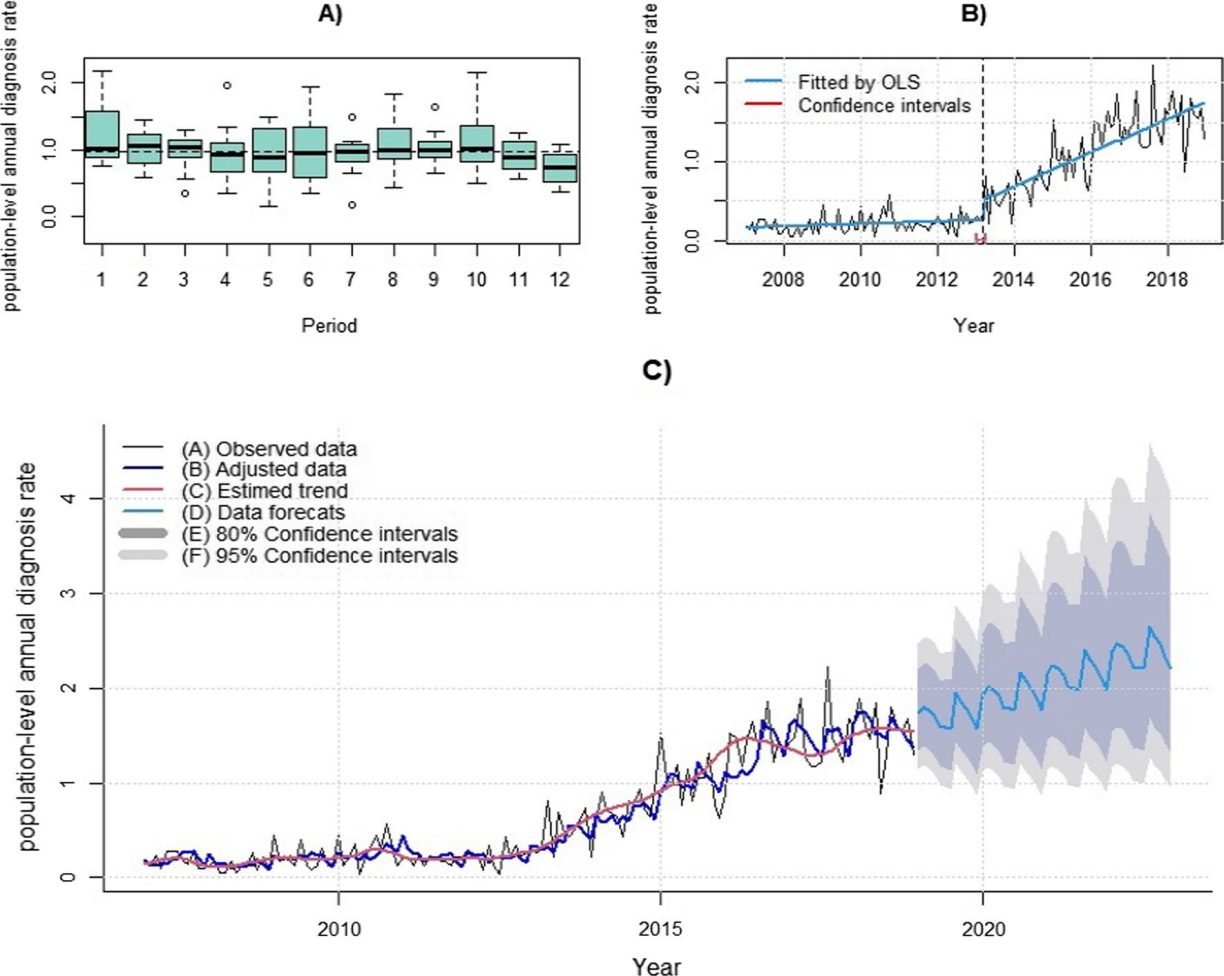
Temporal variability and evolution of the HIV/AIDS population-level diagnosis rates among young MSM in Pará. **A** Monthly HIV/AIDS population-level diagnosis rates; **B** Temporal trend evolution of the HIV/AIDS population-level diagnosis rate between 2007 and 2018; **C** HIV/AIDS HIV/AIDS population-level diagnosis rate forecast from 2019 to 2022

Temporal analysis revealed that the greatest variability in HIV/AIDS population-level diagnosis rates in young MSM from Pará occurred in January, May, June, August, and October (Fig. 2A). Concerning the historical series, the population-level diagnosis rate stabilized from 2007 to March 2013 and the trend increased thereafter (Fig. 2B). The best model to describe the variability of the data was STL + ARIMA (0,1,2). Residual analysis showed a normal distribution (Kolmogorov–Smirnov = 0.04, *p* = 0.65) and variance equality between the observation groups (F = 0.88, *p* = 0.70) and no autocorrelation (Ljung–Box = 31.69, *p* = 0.06; Box–Pierce = 1.66, *p* = 0.99).

The forecast of the behavior of the series from 2019 to 2022 revealed an increasing and oscillating trend with a 38.47% chance of error (MAPE = 38.47, MAE = 0.13, RMSE = 0.18; Fig. 2C).

**Fig. 3:**
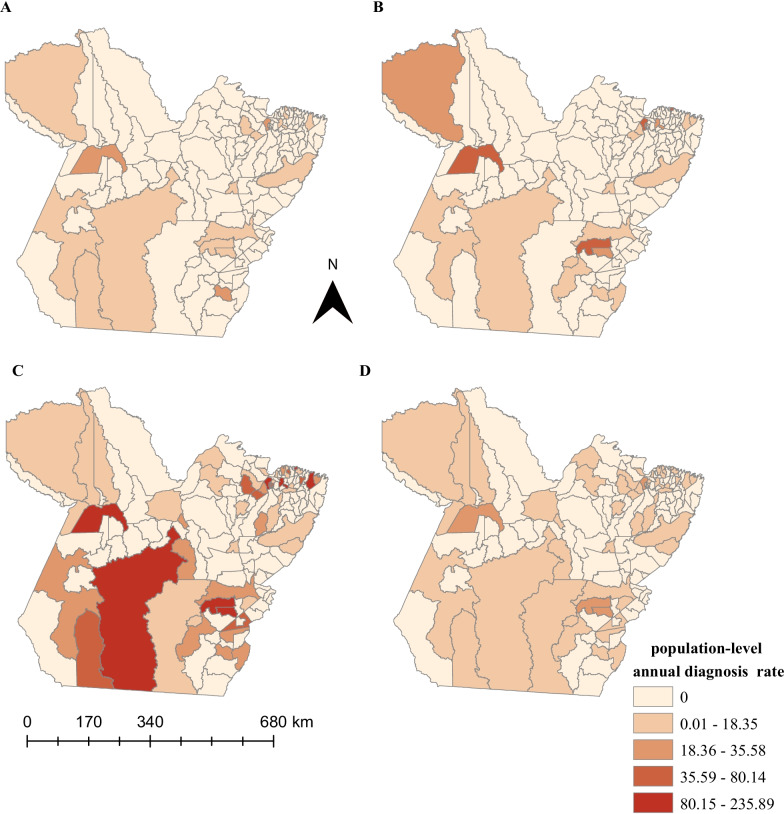
Spatial distribution of the HIV/AIDS HIV/AIDS population-level diagnosis rates among young MSM in Pará. **A** 2007–2010, **B** 2011–2014, **C** 2015–2018, **D** 2007–2018

Figure 3 shows the spatial distribution of the crude HIV/AIDS population-level diagnosis rates for the quadrenniums, 2007–2010 (Fig. 3A), 2011–2014 (Fig. 3B), 2015–2018 (Fig. 3C), and for the 12 years of the study (Fig. 3D). The population-level diagnosis rate increased mainly in municipalities located in the southeast, southwest, and northeast of Pará.

Moran's global analysis did not reveal a spatial autocorrelation in the HIV/AIDS population-level diagnosis rates 2007–2010: *I* =  − 0.06, *p* = 0.26; 2011–2014: *I* = 0.06, *p* = 0.74; 2015–2018: *I* = 0, *p* = 0.97; 2007–2018: *I* = 0, *p* = 0.91).

**Fig. 4:**
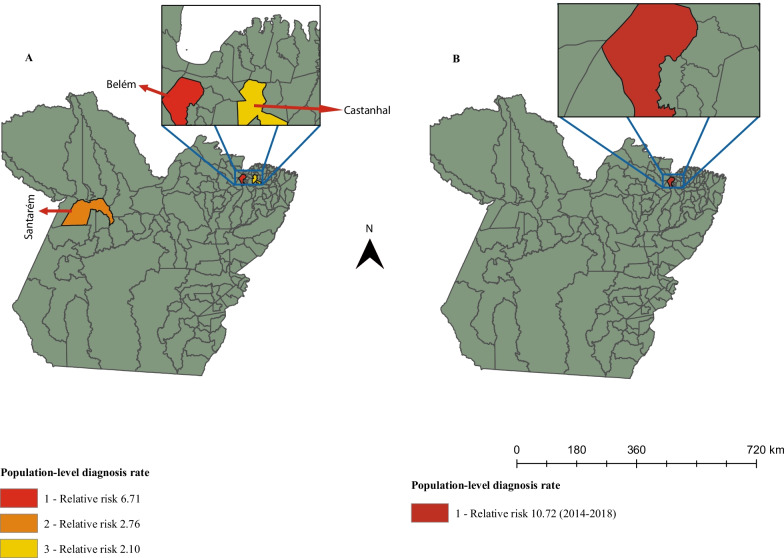
Risk areas for HIV/AIDS among young MSM in Pará. **A** Spatial risk areas, **B** Spatio-temporal risk area

Figure 4A shows the spatial risk analysis, in which three areas at risk of HIV/AIDS were revealed for young MSM. Belém had the highest risk (RR = 6.71, 95% CI 6.16–7, 30, *p* < 0.001), followed by Santarém (RR = 2.76, 95%IC = 2.4–3.18, *p* < 0.001) and Castanhal (RR = 2.1, 95%IC = 1.72–2.56, *p* < 0.001). The spatiotemporal analysis indicated Belém as the only risk zone from 2014 to 2018 (RR = 10.72, 95% CI 9.66–11.55, *p* < 0.001, Fig. 4B).

For the spatial regression, data analysis using the OLS method revealed that “number of young men with formal employment,” “average salary for the formally employed man,” and “percentage of people aged 18 or over with elementary school level” are the best explanatory models (Table 1). Residual autocorrelation analysis did not reveal spatial dependence (*I* =  − 0.01; *p* = 0.97). The GWR method revealed a better fit in the regression analysis than the OLS (GWR: AICc = 659.48; R^2^ = 0.752, adjusted R^2^ = 0.721; OLS: AIC = 727.65, R^2^ = 0.514, adjusted R^2^ = 0.503) and no spatial autocorrelation of residuals (*I* =  − 0.08; *p* = 0.12).

**Table 1:** OLS explicative model to the SDHs impact in HIV/AIDS detection rate in young MSM living in Pará, 2007–2018. Pará, Brazil

Variável	Estimative	Standard deviation	p-valor
Constant	−3.46	0.9	p < 0.001
Number of men with formal jobs	0	0	p < 0.001
Men’s monthly wage average	0.001	0.001	0.087
% of 18 years old people and over with elementary schoolling	0.92	0.025	p < 0.001

R^2^, 0.51/adjusted R^2^: 0.50; AIC, 727.65

**Fig. 5:**
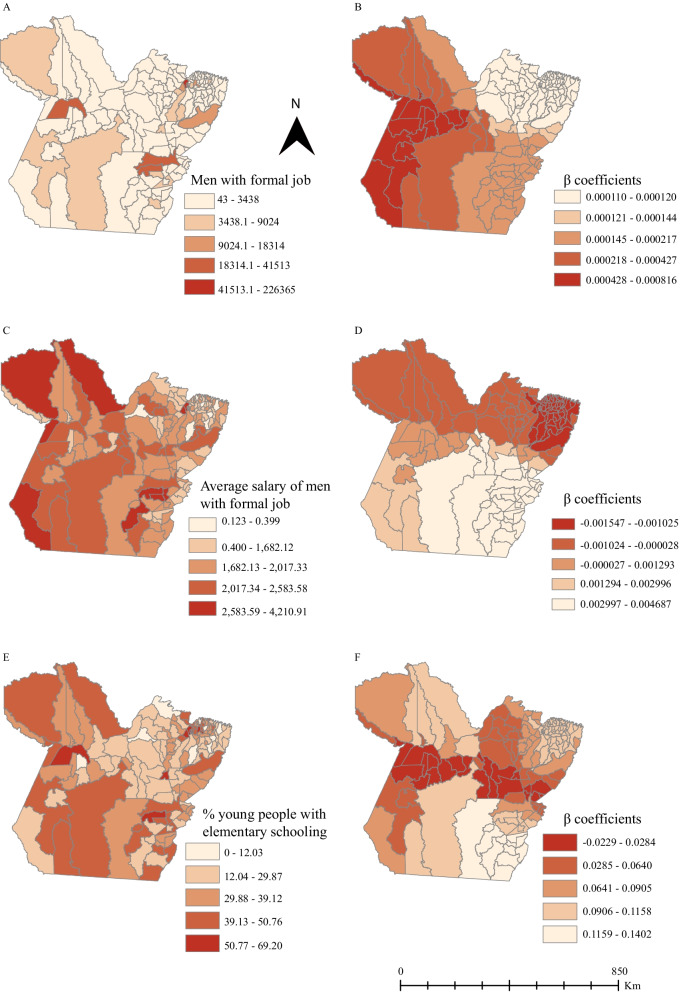
Spatial mapping of the geographically variability of the HIV epidemic in Pará by geografically weighted regression (GWR) promoted by the social determinants of health (SDOH). Spatial distribution of the (**A**) Number of men with formal jobs, **C** Men’s monthly wage average, **E** % of 18 years old people and over with elementar schooling; Coefficients β of (**B**) Number of men with formal jobs, **D** Men’s monthly wage average, **F** % of 18 years old people and over with elementar schooling

Figures 5A, C and E shows the spatial distribution of the independent variables and Figs. 5B, D and F shows the β coefficients of the impact of independent variables on the HIV/AIDS population-level diagnosis rate among young MSM. In the southwest municipalities of Pará, the risk of an increase in the HIV/AIDS population-level diagnosis rates was directly proportional to the number of men with formal employment (Fig. 5B). Regarding the “average salary of men with formal jobs”, the risk of an increase in the HIV/AIDS population-level diagnosis rates was negligible in the north, northeast and west of Pará, where most the municipalities had a small average salary for men with formal jobs (Fig. 5D). The same was noticed to “percentage of young people with elementary schooling” in municipalities in the center, midwest, and middle east of Pará (Fig. 5F).

## Discussion

The results of this study showed that January, May, June, August, and October had the greatest variability in HIV/AIDS population-level diagnosis rates in young MSM in Pará. From March 2013, the increasing trend of the HIV/AIDS population-level diagnosis diagnosis rate is expected to continue until 2022. From 2007 to 2018, the HIV epidemic among young MSM expanded mainly in the southeastern and southwestern municipalities of Pará. Belém, Santarém, and Castanhal had the highest spatial risk for HIV/AIDS. However, Belém was the only municipality to present a spatiotemporal risk between 2014 and 2018. The chance of an increase in the HIV/AIDS diagnosis rate was directly associated with the number of men with formal employment in the southwest of Pará. However, it was negligible in the north, northeast, and west meridionals for the “average salary of men with formal jobs” and for “percentage of young people with elementary schooling” in municipalities in the center, midwest, and middle east of Pará.

The greater variability of HIV/AIDS population-level diagnosis diagnosis rates in January, May, June, August, and December can be explained by the festivities occurring in Pará. January and August follow the holiday season and the July holidays, respectively, where most of the people go to the beaches. In May and June occurs the Feast of Saint Jhon. In October, the catholic event “Círio de Nazaré” is celebrated in Belém, where a great number of tourists come to Belém with an estimative of over 2 million people in the main procession. This suggests a necessity of more preventive interventions against sexually transmissible infections in these months giving access to people to educative and preventive resources.

The trended up temporal behavior of the HIV/AIDS population-level diagnosis rates among Brazilian Amazonian young MSM from March 2013 to 2018 can be due to the expansion of the HIV tests promoted by the testing decentralization in Brazil since 2011. Before 2011, all HIV tests were done only in the Testing and Counseling Centers in Brazil and nowadays the primary healthcare units are also responsible for the tests [21]. However, our results suggest that this decentralization impacted the HIV epidemic among young MSM in Pará only after 2013. From 2013 to 2018 the number of HIV tests received in Pará increased by 373.43% (2013: 229.730 tests; 2018: 857.880 tests) [22].

In addition, the ascending temporal behavior of the HIV epidemic from March 2013 among young MSM can be also due to the compulsory notification of HIV from 2014. Furthermore, in the same year, the Brazilian Health Ministry launched a policy entitled, “Treatment for All,” to eliminate HIV infection by 2030 as proposed by the Joint United Nations Program on HIV/AIDS [23]. This policy expanded HIV test coverage and initiated antiretroviral therapy (ART) immediately after an HIV diagnosis regardless of CD_4_^+^ T-cells count. Although, the continuous increase in the HIV/AIDS population-level diagnosis rate until 2018 suggests a success of the expansion of HIV testing coverage in Pará, other locations that implemented the same policy the HIV population-level diagnosis rate decreased among MSM, as London (8.95%) and Chengdu-China (29%) [24, 25].

In addition, considering the larger territorial extension, all geographic and socioeconomic barriers to people access the healthcare places, such as densely forested areas, with access to urban areas only by boat, rainy climate, and poverty, Pará has minimal resources to fight HIV: in all the state there are only 33 ART-dispensing units, 7 specialized care services, and only 2 pre-exposure prophylaxis (PrEP)-dispensing units [26, 27]. Therefore, it is more than plausible the upward trend of the HIV/AIDS population-level diagnosis rates among MSM since 2013 and its forecasting maintenance until 2022. In central London, since 2015, the HIV population-level diagnosis rate among MSM decreased substantially after expanding and guaranteeing the access of MSM to HIV testing, treatment, and PrEP distribution [25]. In Miami, Florida US, although the HIV incidence rate has decreased among MSM, in the young Latin MSM group it still trending upward as this group have greater difficulty accessing prevention and HIV treatment centers [28].

In the Northern of Brazil, from 2018 to 2019, the number of HIV/AIDS cases among men increase 4.14% (2018: 3190 cases; 2019: 3322 cases) [2]. Although we do not have Brazilian reported regional number of HIV/AIDS cases in men in 2020, the notification of cases in all country decreased 67.34% (2019: 26,141 men cases; 2020: 8434 men cases). Although we do not have a reported regional number of HIV/AIDS cases in men in 2020, the notification of cases in all countries decreased 67.34% (2019: 26,141 men cases; 2020: 8,434 men cases). This decreasing could be due to underreporting cases since the health professionals were relocated to work with the COVID-19 [2]. The population-level diagnosis rate trending up behavior from 2019 to 2022 brings an alert for the possible consequences of the reemergence of conservative politics in Brazil [29] and also of COVID-19 pandemic outbreak affecting the HIV combat policy.

The fact that only Belém, the capital of Pará, had a spatiotemporal risk for HIV between 2014 and 2018 suggests that the strategies to fight HIV have been more efficient in the capital than in other municipalities in the interior. Belém has a higher concentration of healthcare facilities with better access for the people to these services.

The spatial risk for HIV in Belém and Castanhal can be attributed to these cities having the first and fourth highest population densities in the state, respectively. This is in line with what was observed in India. The Indian districts with the highest HIV incidence rates were those with the highest population densities [30]. Santarém is an economic polo and a very touristic municipality in Pará. It attracts tourists from all over the world. Thus, sex tourism might have contributed to the HIV epidemic environment. In China, several HIV risk behaviors were observed among MSM who practiced sex tourism. These behaviors include sexual intercourse without condoms and sex with multiple partners [31]. In addition, another previous study in China showed the municipalities with high density populations and high economic growth had higher HIV population-level diagnosis rates among MSM [14].

The areas with high possibility for an increase in HIV among young MSM in Pará were those in the southern Pará having most of the municipalities with a high number of men with formal jobs, high avarage salary, and high % of men with elementary schoolling level. The souther Pará has a high DHI promoted by a rapid economic rise due to the expansion of the mining industries and hydroelectric constructions and has generated the greatest number of formal jobs in all the state. The young MSM with the best economic conditions can be more exposed to HIV for being allowed to travel abroad and more access to social media technologies, parties, and recreational drugs, for example. A previous study in Vietnam among MSM showed that traveling alone and having a university schooling level was associated with casual sex [32]. In addition, the use of sex-seeking applicatives and recreational drugs in parties is related to an increase in risk to HIV among MSM [33, 34].

Our results showed a greater risk of increasing in the HIV/AIDS population-level diagnosis rates in municipalities associated with the % of young people with elementary schooling. In Pará, on average, the young people have 7.2 years of schooling [35]. To fight HIV among young people, in 2007 the Ministry of Health in Brazil launched the Health at School Program, having as one of the goals the promotion of the sexual health of young people and adolescent students. However, this study suggests a necessity to reinforce even more this policy. In Belém young high school students had a low level of knowledge about HIV and risk behaviors toward the virus, such as the infrequent use of condoms in sexual intercourse and multiple sexual partnerships [36].

The knowledge about HIV deficit can lead to a low HIV risk perception, contributing to the exposition of young MSM to the virus. For example, British MSM showing a low-risk perception of HIV presented more risk behaviors to the virus than MSM showing a high-risk perception [37]. In addition, a previous study comparing MSM behaviors in two different times, 2009 *versus* 2016, showed behavioral changes between the two groups. In 2016, the MSM had a higher schooling level and more condomless receptive and insertive sex with multiple partners, especially those using illicit drugs, with lower knowledge about HIV and counseling uptake [38].

Furthermore, HIV and anti-gay stigmas have also to be fought since they are barrier to MSM looking for HIV services [39]. Consistent with this, a previous study showed a lower acceptance of HIV testing among the MSM living in the Northern of Brazil when compared with other Brazilian regions because of their fear of having a positive result [8].

This study was limited by underreporting and the process of describing variables based on the health professional's judgment. We had to exclude 28 notifications due the missing the name of the municipalities, age, sex, or exposure group. This reinforces the necessity to capacity the health workers to a better quality of the notifications. Additionally, the database made available by the Brazilian Geography and Statistics Institute and Institute for Applied Economic Research was from 2010. Due to the blocking of the financial resource by the federal government, until the present date the new Brazilian census has not yet taken place. Furthermore, in the future census, it is essential to quantify MSM, lesbians, and other population extracts.

In addition, the forecasts up to 2022 did not consider the COVID-19 pandemic outbreak. However, the predictions are always obtained under the premise that there will be no disruptions in the future, and when they occur there are no mathematical models capable of predicting them. As a future study, it is necessary to compare these predictions of this current data with the impact of COVID-19 to see how it changed the prediction course.

## Conclusion

In Brazil, young and MSM populations are considered a priority and a key population in the fight against HIV. However, it is concerning when considering the SDH and the scarcity of policies to combat HIV in Pará. Our study showed an upward trend in the HIV population-level diagnosis rate from 2019 to 2022 signalling an alert to health authorities to reinforce policies against HIV to break this preview. Furthermore, the spatial analysis showing the geographical variability of the HIV epidemic promoted by SHDs provides additional subsidies to focal and more efficient policies.

Public policy against HIV will not be successful if people do not have access to sexual health education, prevention, testing, or HIV treatment. Social equality and a guarantee of basic human rights to all citizens can prevent the 2022 forecasted increase in the HIV/AIDS population-level diagnosis rate.

## Data Availability

The datasets analysed during the current study are not publicly available due restrictions apply to the availability of these data. All data in this study were used under license, and so are not publicly available.

## Abbreviations

AIC: Akaike information criterion
AICC: Corrected Akaike information criterion
AIDS: Acquired immunodeficiency syndrome
ARIMA: Autoregressive integrated moving average
CI: Confidence interval
DHI: Development human index
GWR: Geographically weighted regression
HIV: Human Immunodeficiency Virus
LISA: Local indicator of spatial autocorrelation
MSM: Men who have sex with men
OLS: Ordinary least squares model
PrEP: Pre-exposure prophylaxis
SDOH: Social determinants of health
STLF: Locally weighted polynomial regression
